# What is all the noise about interval timing?

**DOI:** 10.1186/1471-2202-14-S1-P44

**Published:** 2013-07-08

**Authors:** Sorinel A Orisan, Catalin V Buhusi

**Affiliations:** 1 Department of Physics and Astronomy, College of Charleston, Charleston, SC 29424, USA; 2 Department of Psychology, Utah State University, Logan, UT 84322, USA

## 

Interval timing (IT) is an essential capability of living organisms from invertebrates to humans that allows them to accurately estimate durations in the range of seconds to minutes. In peak interval procedure [1,2] subjects receive reinforcement for the first response that occurs after some criterion time (CT) has elapsed. After the CT is learned, the subject is given reinforced trials alternating with probe trials in which the to-be-timed stimulus is presented for three times the CT without reinforcement. Under these conditions, the maximal rate of responding (output function) is found to occur at the original CT [2]. Behavioral experiments showed that that the standard deviation of the output function is proportional to the CT [1,3]. Both lesion and pharmacological studies indicated the involvement of cortico-striato-thalamic circuits in IT, including premotor and supplementary motor areas, frontal operculum and dorsolateral prefrontal cortex, striatum and its afferent projections from the substantia nigra pars compacta [4].

We implemented a minimal Striatal Beat Frequency (SBF) model [5,6], that includes: (1) an oscillator block (OSC), presumably localized in the prefrontal cortex area; (2) a memory block (MEM), presumably associated with the nucleus basalis magnocellularis, frontal cortex and/or hippocampus or fimbria fornix, that stores information about the state of the brain at the moment of reinforcement; (3) a neuromodulator block that mimics primarily the modulation of cortical or thalamic (glutamate) induced striatal spiny neuron activity, and the threshold for coherent activity detection due to dopamine release from substantia niagra pars compacta; and (4) a decision block presumably associated with the striatal spiny neurons integrating a very large number of different inputs, and responding selectively to particular reinforced patterns.

The states of all OSC neurons determine the vector of state that is received by each spiny neuron together with the vector of state of OSC stored in MEM at CT. In our implementation, the output is generated through coincidence detection, i.e., dot product, between the current state of OSC and the state of OSC stored in MEM at CT. Biological noise induces different types of variabilities, e.g., random fluctuation in the frequencies of OSC neurons, errors in storing/retrieving the criterion time in MEM, etc. We investigated both analytically and numerically the properties of the output function generated by the SBF model and found that: (1) in the absence of any variability in the parameters of the SBF model, the width of the output function that measures the spread of behavioral responses is constant, therefore, violating the scalar property, (2) if variability is allowed, for example in the memorization/retrieval of CT, then the output function of SBF model is always Gaussian, which is a consequence of the central limit theorem, regardless of the probability distribution function (pdf) of fluctuating parameter. Moreover, we found that the scalar property is also preserved regardless of the pdf of fluctuating parameters.
